# Plasma-Treated Poly(Lactic Acid): Deciphering the Structure of a Versatile Engineering Material

**DOI:** 10.1021/acsomega.5c08696

**Published:** 2025-12-09

**Authors:** Adrián Fontana-Escartín, Nicolas Simon, Oscar Bertran, Alessandro Contini, Juan Torras, Carlos Alemán

**Affiliations:** † IMEM-BRT Group, Departament d’Enginyeria Química, EEBE, 16767 Universitat Politècnica de Catalunya, C/Eduard Maristany, 10-14, 08019 Barcelona, Spain; ‡ Barcelona Research Center in Multiscale Science and Engineering, EEBE, Universitat Politècnica de Catalunya, C/Eduard Maristany, 10-14, 08019 Barcelona, Spain; § Departament de Física EETAC, Universitat Politècnica de Catalunya, c/Esteve Terrades, 7, 08860 Castelldefels, Spain; ∥ Dipartimento di Scienze Farmaceutiche − Sezione di Chimica Generale e Organica “Alessandro Marchesini”, 9304 Università degli Studi di Milano, Via Venezian, 21, 20133 Milano, Italy; ⊥ Institute for Bioengineering of Catalonia (IBEC), The Barcelona Institute of Science and Technology, Baldiri Reixac 10-12, 08028 Barcelona, Spain

## Abstract

Conventional thermoplastics treated with plasma under controlled conditions have proven to be low-cost materials with advanced properties that are highly useful in engineering in applications ranging from food packaging to the electrochemical detection of bioanalytes. However, the changes induced by plasma in their chemical structures are still relatively unknown. In this work, we address the study of these materials, focusing on poly­(lactic acid) (PLA), which is electrochemically inert but, once treated with low-pressure oxygen plasma, is capable of detecting bioanalytes such as dopamine based on the acquired electrochemical response. More specifically, we have studied the chemical structure of PLA treated with different low-pressure plasmas using X-ray photoelectron spectroscopy, in-depth micro-Raman, ζ-potential, electrical resistance, and contact angle measurements, proving also the performance of this material as an electrochemical sensor for detecting dopamine. In addition, we performed atomistic molecular dynamics simulations to compare the structural properties of plasma-treated PLA with those of amorphous and crystalline PLA. The results revealed the formation of specific functional groups at depths up to 10 μm and showed variations in the material’s electrical properties as well. Additionally, simulation studies revealed that the structure of plasma-treated PLA differs from those of amorphous and crystalline PLA in terms of interactions and conformational order/disorder.

## Graphical abstract

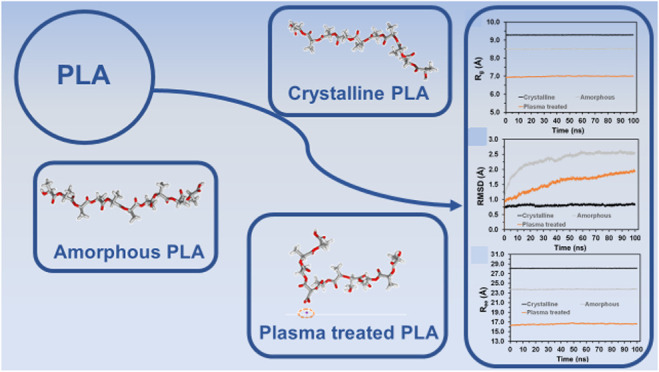

## Introduction

Plasma treatment technologies are used to alter the surface properties of a wide range of materials, making them cleaner, more hydrophilic/hydrophobic, and easier to bond and/or adhere.^1–5^ Plasma, which is described as the fourth aggregated state of matter, is a partially or fully ionized gas that contains electrons, a collection of positively and negatively charged ions, and both neutral and excited gas atoms and molecules.^6,7^ In recent years, this surface modification technique has been considered very advantageous in terms of economy (simple operation), efficiency (less time), and environmental friendliness (no chemicals and no generated waste).^1–7^

Plasmas are typically divided into two broad categories: thermal plasma and nonthermal (or cold) plasma. While in thermal plasma a significant proportion of gas molecules/atoms are ionized by applying thermal energy (*i*.*e*. both ionized electrons and species have the same energy in each degree of freedom), in cold plasma (or nonequilibrium plasmas) electrons are selectively energized, typically by a strong electric field, causing electrical breakdown of the gas species (i.e. electrons heat up rapidly due to their low mass while gas species remain cold). Accordingly, cold plasma is easier to handle, relatively safer, environmentally friendly, and cheaper compared to thermal plasma.

Cold plasma technologies can be grouped into two categories: atmospheric pressure plasma and low-pressure plasma. Atmospheric cold plasma is produced at standard atmospheric conditions (i.e. without a vacuum chamber and pumps to maintain pressure) using dielectric barrier discharge, plasma jet, gliding arc discharge, or corona discharge, among other technologies.^8–10^ In low-pressure cold plasma, ionized species are precisely generated in a vacuum chamber at reduced pressure, which is maintained by a pumping unit that clears unwanted gases from the system, using electrodes that can be powered by electromagnetic generators using direct current (DC), alternating current (AC), radio frequencies, or microwave frequencies. The advantages and drawbacks of atmospheric and low-pressure cold plasmas have been extensively reviewed.^11–13^

Independent of the pressure, the cold-plasma-induced chemical and physical effects depend on the gas (e.g., O_2_, N_2_, air, and Ar/H_2_ mixtures) and excitation energy used for plasma formation.^14–16^ For example, O_2_ and N_2_ gases are used to immobilize carboxyl and amine groups on the surface, respectively, while air is used for the immobilization of both polar groups. Cold plasma-modified surfaces have caught the attention of many interdisciplinary scientists for tethering (bio)­molecules using such immobilized carboxyl and/or amine groups.^17–20^ The nonthermal plasma surface modification technique has evolved as a very promising candidate for biomedical applications.

Cold plasma offers several potential applications in the food and biomedical industries. Food decontamination,^21,22^ food quality improvement,^23,24^ toxin degradation,^25,26^ and surface modifications of packaging materials are among the main applications of cold plasma for the food industry,^27,28^ while new horizons have been opened in the biomedical field with applications like sterilization,^29^ blood coagulation,^30,31^ wound healing,^32^ skin disinfection,^33^ and cancer therapy.^34,35^

Despite all such opportunities, a new application of cold plasma, which can be extended to many fields, is receiving attention. This is the utilization of such technology to enhance and promote the electrical response of materials.^36^ For example, the electrical properties of MXene-coated textiles and HfAl_2_O_*x*_ were enhanced using a cold plasma treatment with Ar and a mixture of Ar and O_2_, respectively.^36–41^ Besides, O_2_ plasma has been used to alter the molecular structure of single-walled carbon nanotube (SWCNT) films,^38^ ZnO nanoflowers,^39^ and graphene sheets,^40^ improving their electrochemical response. On the other hand, the electrical response of soft materials, like for example polymers, has also been modified using cold plasma treatments.^41^ In a very recent study, the ionic conductivity of polytetrafluorethylene (PTFE) substrates was promoted using NH_3_ and O_2_ cold plasma treatments, which were used to propose PTFE-based binders for anion exchange membranes in water electrolysis cells.^41^ Also, a plasma approach, but using very well-controlled operation conditions, was employed to transform dielectric polymers into electro-responsive ones.^42–45^ Although the treatment of thin films (two-dimensional (2D) samples) of dielectric polymers, such as polyethylene and polypropylene, yields an effective electrical response, it also compromises the mechanical integrity of such structures.^42,43^ This structural drawback was overcome by applying a low-pressure O_2_ plasma treatment to three-dimensional (3D) printed samples.^44,45^ This strategy allowed us to produce electrochemical sensors for biomolecules using dielectric biopolymers, such as polylactic acid (PLA).

In this work, we present a detailed study of the structural properties of PLA treated with low-pressure O_2_ plasma, which have not been investigated before. For this purpose, 0.4, 0.6, and 0.8 mbar of O_2_ were introduced inside the evacuated chamber with 3D-printed PLA samples to create controlled energetic plasmas. After a production time of 2 min, the obtained plasma-treated samples were analyzed by X-ray photoelectron spectroscopy (XPS), micro-Raman spectroscopy, and electric and contact angle measurements. In addition, its performance as an electrochemical sensor was validated by detecting dopamine (DA). Finally, atomistic molecular dynamics (MD) simulations were conducted on amorphous, crystalline, and plasma-treated PLA to ascertain the structural differences among the three systems.

## Methods

### Preparation of Plasma-Treated PLA Samples

PLA discs (1.0 cm diameter) were obtained using a BCN3D SIGMAX R19 3D printer at 230 °C employing a nozzle with 0.3 mm diameter (infill density at 100%). The PLA filaments were supplied by MCPP Netherlands BV.

PLA discs were placed inside a metallic holder in the chamber with a volume of 2.6 dm^3^, and the whole system was purged under vacuum and filled with O_2_ gas until the desired pressure (0.4, 0.6, or 0.8 mbar) was reached. The O_2_ pressure was automatically regulated by a valve, using a minimum gas flux (∼20 sccm), to ensure the target final pressure. Then, an electric discharge was applied by employing a generator with a maximum radiofrequency of 13.56 MHz and a maximum power output of 300 W. The plasma was created by using a 100 W power supply for 2 min. Samples were stored under a vacuum until use.

### Experimental Characterization

#### X-ray Photoelectron Spectroscopy

Analyses were performed on a SPECS system (SPECS Surface Nano Analysis GMbH, Berlin, Germany). Spectra were acquired in ultrahigh vacuum (5.0 × 10^–9^ mbar) with an XR50 Al anode source operating at 150 W and a Phoibos 150 MCD-9 detector (D8 advance, SPECS Surface Nano Analysis GmbH, Berlin, Germany). The spectra were recorded at a pass energy of 25 eV with an energy step size of 1.0 eV for survey spectra and 0.1 eV for high-resolution spectra. C 1s peak was used as a reference, fixing it at 284.8 eV. Since the samples did not show significant peak deformation during the experiment, no further charge compensation was needed. Casa XPS software (Casa Software Ltd., Devon, U.K.) was used for the determination of atomic elemental composition while applying the manufacturer’s set of relative sensitivity factors.

#### Micro-Raman

Raman spectra were obtained using the inVia Qontor confocal Raman microscope (Renishaw), equipped with a Renishaw Centrus 2957T2 detector and a 785 nm laser. Raman analyses at different depths within the samples were performed. To improve depth resolution, a laser power of 1% and an exposure time of 10 s were employed. The spectra were recorded within the Raman shift range of 600–4000 cm^–1^, with depths of 1, 5, and 10 μm.

#### ζ-Potential and Resistance

Measurements of pristine and plasma-treated PLA plates (20 mm × 10 mm × 1 mm) were performed using a SurPass 3 Electrokinetic Analyzer by Anton Paar. An adjustable gap cell (20 mm × 10 mm) was used for all measurements. The PLA plates were dried before being placed in the cell, and a neutral pH (∼7) electrolyte solution of KCl was used. Measurements were carried out at room temperature (∼22 °C). The SurPass software controlled the experimental parameters and processed the data, ensuring precise and reproducible results. Three measurements were taken to confirm the consistency.

#### Density

A rectangular PLA sample was 3D printed with dimensions of 10 mm × 10 mm × 0.5 mm and a 100% infill density. Samples were weighed using a Sartorius Quintix125D-1S balance, which offers a high resolution of 0.01 mg, both before and after oxygen plasma treatment. The plasma treatment was conducted at varying pressures of 0.4, 0.6, or 0.8 mbar. Each experimental condition was replicated three times to ensure statistical reliability.

#### Contact Angle

CA measurements were conducted by using the sessile drop method at room temperature. Images of Milli-Q water, 75:25 Milli-Q water: ethanol (EtOH) and 50:50 Milli-Q water: ethanol drops (0.5 μL) were recorded after stabilization (∼10 s) with a DSA25S (Krüss GmbH) and analyzed using the Advance (Krüss) software. For each sample, the average CA value and the corresponding standard deviation were derived from at least ten independent measures.

#### Detection of Dopamine

The electrochemical detection of dopamine (DA) was studied by cyclic voltammetry (CV) using a three-electrode cell. The latter consisted of the plasma-treated PLA plasma samples as the working electrode, Ag|AgCl (KCl, 3M) reference electrode, and platinum wire as the counter electrode. The electrolyte employed was a phosphate-buffered saline (PBS) solution (pH 7.4). The scan rate was 50 mV/s, while the potential interval ranged from 0.50 to 0.50 V. Measurements were performed by adding different concentrations of DA to the PBS solution.

### Computer Simulations

#### Molecular Models

Three different PLA states were considered: amorphous, crystalline, and plasma-treated. MD simulations for each state were conducted on systems formed by 180 polymer chains, with each one containing 10 repeating units.

For the amorphous model, PLA chains were placed randomly in a box with dimensions (80 Å × 80 Å × 80 Å) using Packmol^46^ with a minimum interatomic distance of 2.0 Å to prevent overlaps. The density of such a generated starting structure was 0.652 g/cm^3^. For the crystalline model, all chains were arranged in a helical conformation that was generated using the atomic coordinates reported for the crystal structure of the α-form.^47^ The unit cell parameters (*a* = 10.6 Å, *b* = 6.1 Å, *c* = 28.8 Å) were replicated in three dimensions to construct the crystal with a final system size of 52.675 × 55.530 × 57.676 Å^3^. Finally, the plasma-modified model was obtained by modifying two or three randomly chosen residues of each polymer chain in the amorphous model. More specifically, 144 chains were modified at two residues as follows: in one of such residues, the methyl group was changed by a hydroxymethyl group (i.e. CH_3_ by CH_2_OH), while in the other, the methyl group was changed by a carboxylate group (i.e. CH_3_ by COO^–^). On the other hand, 36 chains were modified at three residues as follows: in 18 chains, three methyl groups were changed by two hydroxymethyl and one carboxylate groups, while in the other 18 chains, three methyl groups were changed by one hydroxymethyl and two carboxylate groups.

#### Force-Field Parameters

Stretching, bending, torsional, and van der Waals force-field parameters for PLA residues were taken from the second generation of the general Amber force field (GAFF2).^48^ The atomic charges for each segment of the polymeric chain (head, repeat unit, and tail) were calculated applying the restrained electrostatic potential (RESP) methodology.^49^ For this purpose, geometry optimization was done on model molecules at the HF/6–31G­(d) level to determine the lowest energy conformation, using the Gaussian09 software.^50^ ESP was computed at the same level on these conformers, and the results were then used to determine the RESP charges of each segment (Figure S1).

#### Simulations Protocol

MD simulations were performed using the Amber20 package.^51^ The temperature and pressure were controlled via Berendsen thermostat and barostat, respectively.^52^ For the amorphous and plasma-treated systems, the isotropic barostat was used, while the anisotropic barostat was used for the crystalline system. Before the production of MD trajectories, a quenching process was performed for the amorphous and the plasma-treated systems.^53^ First, systems were minimized using the steepest descent method to reduce atomic clashes and correct the distances. After that, the amorphous and plasma-treated systems were heated to 600 K to obtain PLA in a liquid state and allow an accelerated reorganization of the polymeric chains. The systems were then equilibrated over 1 ns in an isothermal–isobaric (NPT) ensemble. The systems were then cooled to 298 K with a quenching rate of 20 K/ns in an NPT ensemble to the experimental conditions. The quenching simulated time resulted in a 15 ns procedure. After that step, a 1 ns NVT equilibration was performed, and a 10 ns NPT simulation was performed to obtain the equilibrium density value and the initial structure for the production runs. After the systems were equilibrated, the production simulations were performed for 100 ns with a time step of 2 fs. The temperature was set at 298 K by using the Berendsen thermostat, and the systems were kept in the NVT ensemble. For the crystalline system, the production run was started after the steepest descent minimization.

## Results and Discussion

Figure 1 shows representative XPS survey spectra of pristine and plasma-treated PLA, which were used to determine the chemical compositions of the samples (Table 1). The XPS spectrum of pristine PLA reveals two main contributions corresponding to C 1s at ∼285 eV and O 1s at ∼532 eV, attributable to the PLA’s chemical composition. The low-pressure O_2_ plasma treatment caused the incorporation of new functional groups on the PLA surface. As expected, this phenomenon was more pronounced with an increasing O_2_ pressure. The functionalization, clearly generated by oxygen-rich species, increased the intensity of the O 1s peak, while the C 1s peak decreased considerably. As a result, the C 1s/O 1s atomic ratio decreased from 3.1 for pristine PLA to 2.8, 2.4, and 2.2 for samples treated using 0.4, 0.6, and 0.8 mbar O_2_ plasmas, respectively (Table 1).

**Figure 1:**
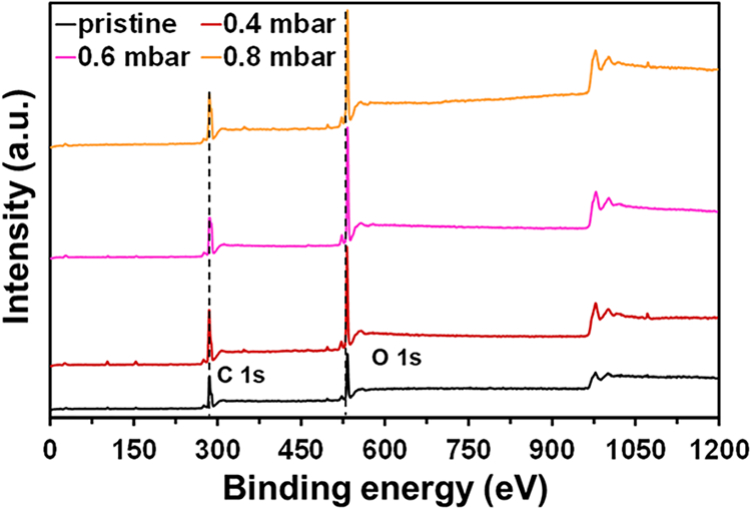
XPS survey of pristine and plasma-treated PLA samples.

**Table 1:** Atomic Percent Composition (C, O, and N; in %) of Pristine and Plasma-Treated PLA Samples as Determined by XPS^a^

sample	C	O	N	C/O
pristine	75.8	24.0	0.2	3.1
0.4 mbar	73.8	26.0	0.2	2.8
0.6 mbar	70.4	29.3	0.3	2.4
0.8 mbar	69.1	30.6	0.3	2.2

a The C/O atomic ratio is also indicated.

The split-peak fit of C 1s showed that the carbon atoms of pristine PLA (Figure 2) have three peaks of different energies (i.e. O–C=O, O–C–O, and C–C at 288.9, 286.8, and 284.8 eV, respectively). The contribution of the O–C=O and O–C–O bonds was similar and 2.7 times lower than that of the C–C bond (Table 2). Such three peaks were maintained with similar contributions for the sample treated using 0.4 mbar O_2_ plasma (Figure 2). However, the sample treated using 0.6 mbar O_2_ plasma (Figure 2) showed a significant reduction in the contribution of the C–C bond (−15.9%), whereas the contribution of the O–C–O and O–C=O bonds increased considerably (+7.4 and +8.5%, respectively). This phenomenon was much more pronounced for the sample treated using 0.8 mbar O_2_ plasma (Figure 2) that, in addition, showed two new peaks at 285.4 and 282.8 eV, attributed to the reaction with the atoms of the metallic holder (C–M in Table 2). The most striking feature is that the atomic ratio between C–C: O–C–O and C–C: O–C=O decreased from 2.7 (pristine) to 0.9 (0.8 mbar).

**Figure 2:**
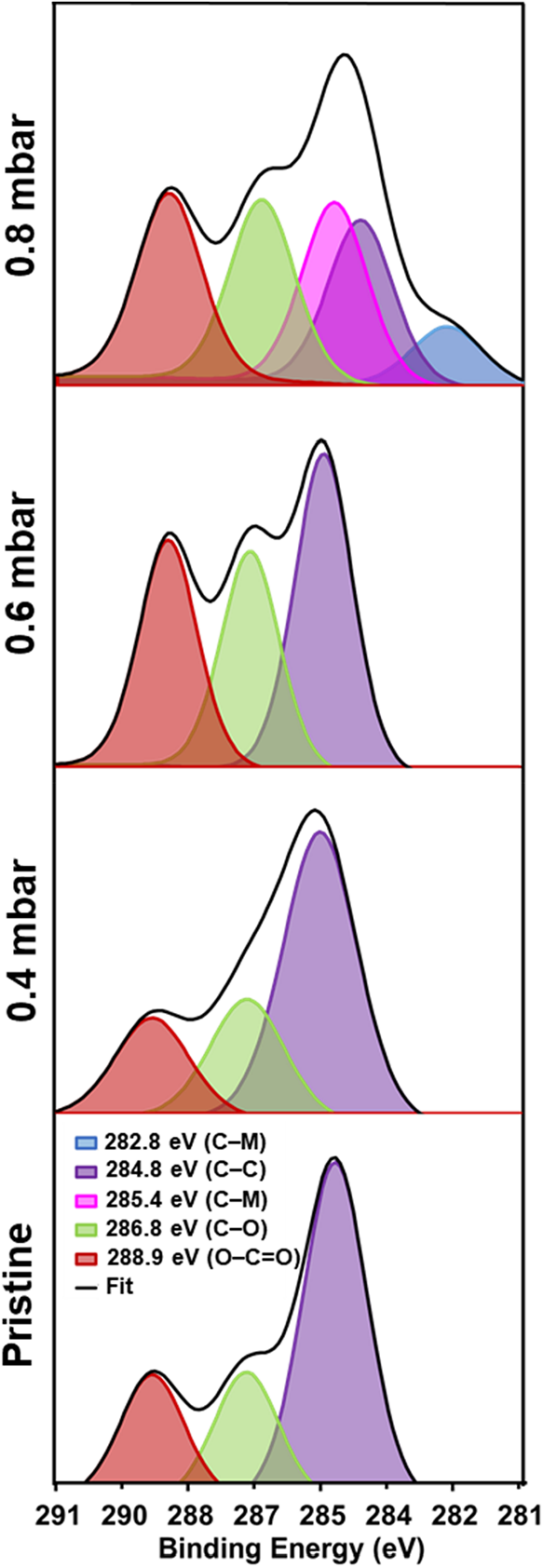
High-resolution region of C 1s and decomposed peaks for pristine and plasma-treated PLA samples, as determined by XPS.

**Table 2:** Contribution of the Different Species to the C 1s and O 1s Peaks as Determined by Decomposing the Corresponding Peaks

	pristine	0.4 mbar	0.6 mbar	0.8 mbar
Split-peak fit of C 1s				
C–C (284.8 eV)	57.7	57.0	41.8	21.2
O–C–O (286.8 eV)	21.2	23.5	28.6	23.2
O–C=O(288.9 eV)	21.1	19.5	29.6	23.7
C–M (282.8 eV)	-	-	-	8.6
C–M (285.4 eV)	-	-	-	23.3
Split-peak fit of O 1s				
C=O(532.1 eV)	56.2	82.8	55.6	49.2
C–O (533.5 eV)	43.8	17.2	44.4	40.0
O–M (527.5 eV)	-	-	-	4.0
O–M (529.7 eV)	-	-	-	6.8

The split-peak fit of the O 1s bond (Figure S2) was fully consistent with the previous observations, which showed two contributions at around 532 and 534 eV assigned to the C=O and C–O bonds.^54^ The ratio between C=O/C–O signals of PLA in this work is 1.3 (Table 2). This ratio increased to 4.8 for the sample treated using 0.4 mbar O_2_ plasma (Figure S2, Table 2), while it decreased to 1.2 for the samples treated using 0.6 and 0.8 mbar O_2_ plasma (Figure S2, Table 2). These variations, together with the chemical compositions displayed in Table 1, indicate that C=O-containing species are preferentially formed at an O_2_ pressure of 0.4 mbar, while C–O-containing species (i.e. hydroxyl groups) are favored at 0.6 and 0.8 mbar. As observed before for the C 1s, the highest O_2_ pressure also favors the formation of C–M bonds with the atoms of the metallic holder. It is worth noting that the incorporation of oxygen-containing functional groups was also achieved using air plasma^42^ but not with N_2_ and Ar plasmas.^54,55^

Exhaustive inspection of pristine and plasma-treated PLA samples was performed by in-depth Raman spectroscopy measurements (i.e. at the surface, and at depths of 1, 5, and 10 μm). Figure 3a displays the normalized Raman spectra (844 cm^–1^, associated with the O–C=O in-plane bending) measured at the surface. Pristine PLA shows the characteristic fingerprints: C–H stretching modes of CH_3_ (3000–2887 cm^–1^), C=O stretching (1772 cm^–1^), asymmetric and symmetric CH_3_ deformation modes (1446 and 1301 cm^–1^, respectively), asymmetric C–O–C (1188 cm^–1^), CH_3_ asymmetric groups (1128 cm^–1^), C–CH_3_ stretching (1044 cm^–1^), C–COO vibrations (874 cm^–1^), and O–C=O in plane bending (844 cm^–1^).^56^ Raman spectra recorded for the different plasma-treated samples were similar to those of pristine PLA, even though there was a change in the intensity of the vibrations associated with the C–H stretching modes of CH_3_, C=O stretching, asymmetric CH_3_ deformation mode, and C–COO vibrations. These variations are illustrated in Figure 3b, which displays how the intensity of such bands varies for plasma-treated PLA relative to pristine PLA. The intensity of C–H stretching modes of CH_3_ decreases by 5–19%, while those of C=O stretching and C–COO vibrations increase by 1–9 and 1–2%, respectively, regardless of the plasma treatment. Instead, the intensity of the asymmetric CH_3_ deformation mode increases or decreases depending on the O_2_ pressure. These results clearly indicate the existence of chemical changes on the surface of plasma-treated samples.

**Figure 3:**
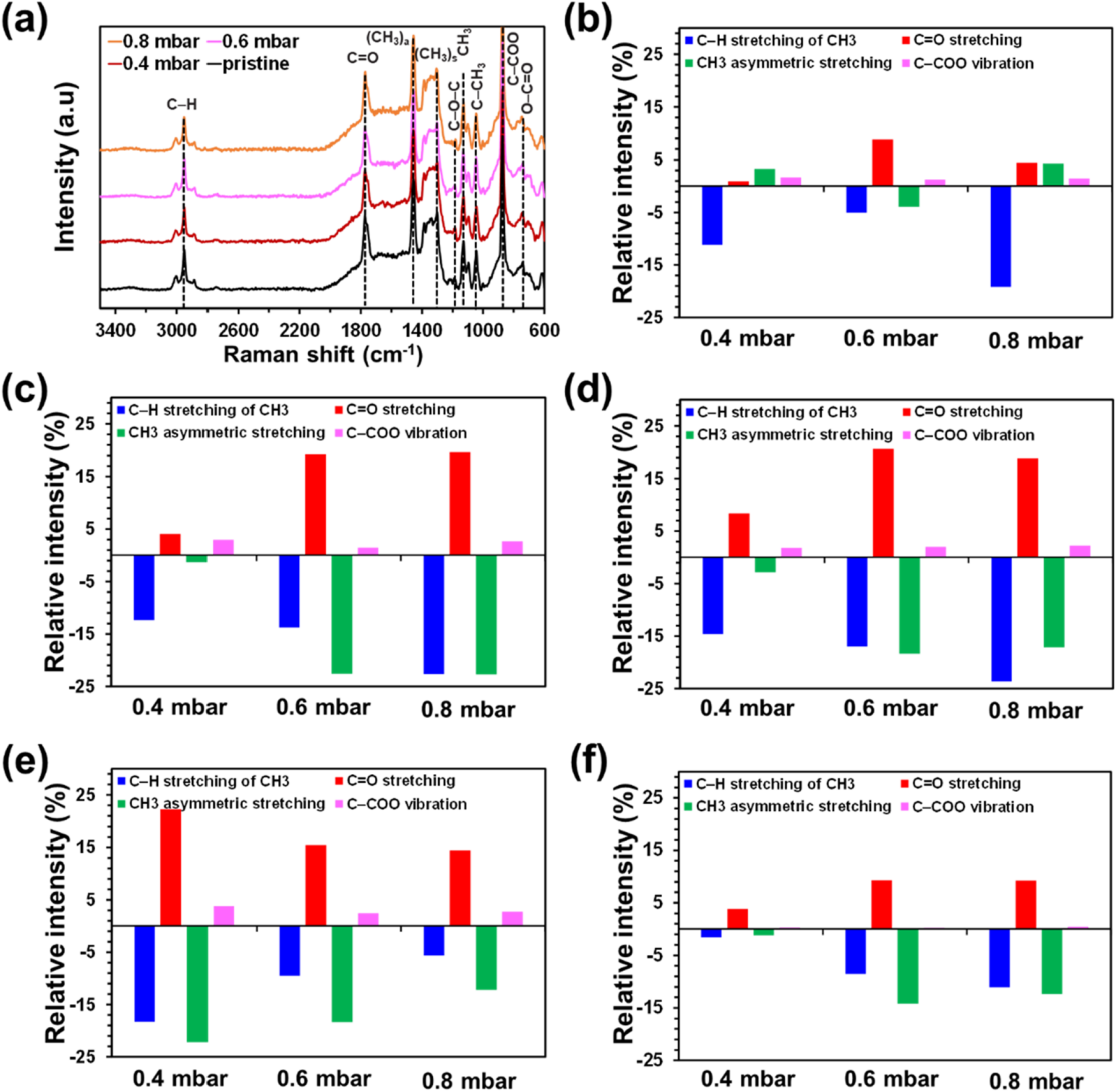
(a) Raman spectrum of pristine and plasma-treated PLA samples at the surface (spectra recorded at 1, 5, and 10 μm depths are shown in Figure S3). (b–e) Variation of the intensities of the C–H stretching modes of CH_3_, C=O stretching band, asymmetric CH_3_ deformation mode, and C–COO vibrations for plasma-treated PLA relative to pristine PLA at the surface (b) and at depths of 1 μm (c), 5 μm (d), and 10 μm (e). (f) Variation of the same bands for pristine PLA at depths of 1, 5, and 10 μm relative to pristine PLA at the surface.

Raman spectra recorded at 1, 5, and 10 μm depths are displayed in Figure S3. Figure 3c–e shows the variation of the intensity of the bands mentioned above in plasma-treated PLA relative to pristine PLA at the same depths. It is worth noting that the intensity of the bands shows higher variations at the different depths than at the surface, which suggests that the plasma treatment reaches a depth of at least 10 μm. In order to confirm such a hypothesis, the variation of the studied bands has been analyzed for pristine PLA. More specifically, Figure 3f compares the relative variation of the intensity of the above-mentioned bands for pristine PLA at depths of 1, 5, and 10 μm with respect to pristine PLA at the surface. As it can be seen, the intensity of the C–H stretching modes of CH_3_, C=O stretching, and asymmetric CH_3_ deformation mode at depths of 5 and 10 μm shows some variations with respect to the surface spectrum, which has been attributed to the 3D printing process (i.e. the migration of some components of the filament, as for example plasticizers and pigments, through the layers). However, such variations are significantly lower than those found for plasma-treated samples at the same depths. Overall, these results confirm that the applied plasma treatment affects up to a depth of at least 10 μm. Furthermore, Raman spectroscopy results are consistent with XPS spectra, showing that plasma-treatment enhances the formation of oxygen-containing groups. Our hypothesis is that the atomic ionized species forming the plasma diffuse to a certain depth because of the plasma-induced surface alterations, as reported in a recent morphological study.^16^

The ζ-potential (ZP) averaged values (*n* = 5) of pristine and plasma-treated PLA samples are listed in Table 3. As can be seen, the ZP increases linearly with the pressure of O_2_ in the plasma treatment (Figure S4), evidencing the increasing formation of negatively charged groups, in agreement with XPS data. These results are opposite to those reported by Wheatley and co-workers,^57^ who applied O_2_ plasma to PLA ultrasound contrast agents. More specifically, those authors showed that the ZP decreased in an absolute value with both plasma power and plasma exposure time. However, these trends are counterintuitive to what would be expected from the generation of oxygen-containing polar groups, being a surprise for the authors themselves.^57^

**Table 3:** ζ-Potential and Static Resistance Obtained for Pristine and Plasma-Treated PLA

	pristine	0.4 mbar	0.6 mbar	0.8 mbar
ZP (mV)	–11.7 ± 0.6	–18.3 ± 0.3	–20.6 ± 0.9	–25.2 ± 0.6
*R*_s_ (kΩ)	198.9 ± 5.2	170.4 ± 1.8	129.7 ± 1.3	150.4 ± 0.9

On the other hand, Table 3 shows that the static resistance (*R*_s_) of PLA, which corresponds to the normal Ohmic resistance, also decreases upon plasma treatment. In this case, however, no linear correlation was observed between *R*_s_ and the O_2_ pressure, likely due to the plasma-induced chemical changes inside the samples. Thus, the *R*_s_ was higher for the sample treated at 0.8 mbar O_2_ pressure than for the one processed at 0.6 mbar, which is consistent with the variation of the Raman bands at a depth of 10 μm (Figure 3e). More specifically, the slightly greater changes observed in samples treated at 0.6 mbar compared to 0.8 mbar explain the higher ionic transport and, therefore, the lower *R*_s_ of the former.

The CA was determined for untreated samples as well as for PLA treated with O_2_ plasma at 0.8 mbar using water, 75:25 water: EtOH and 50:50 water:EtOH. Results, which are compared in Figure 4, indicate that plasma-treatment drastically decreases the hydrophobicity of PLA, with the water CA decreasing 33°. This difference decreases to 24° when the 75:25 and 50:50 water: EtOH mixtures were employed, evidencing the polar nature of the generated functional groups.

**Figure 4:**
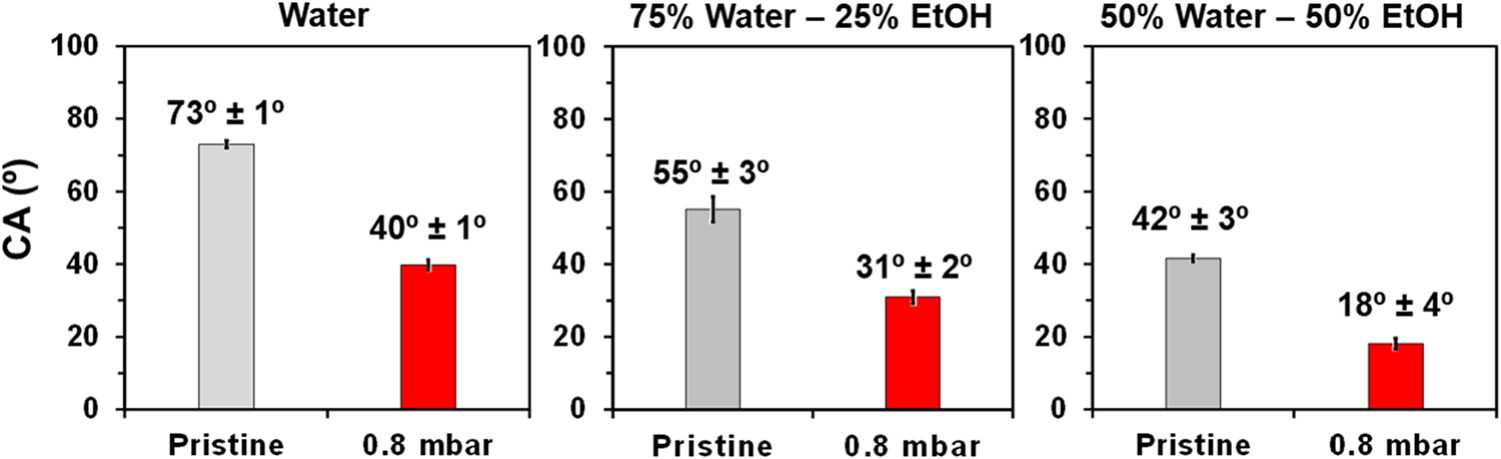
Contact angle (CA) values determined using water: 75:25 water: EtOH and 50:50 water: EtOH for untreated PLA and plasma-treated PLA (0.8 mbar).

Finally, although the performance of plasma-treated thermoplastic as electrochemical sensors has been previously reported,^16,44^ we have conducted additional assays for the detection of DA using PLA treated with O_2_ plasma at 0.8 mbar as the working electrode. For this purpose, detection experiments were conducted using CV and considering an increasing amount of DA. Figure 5a displays the cyclic voltammograms recorded in PBS without DA and after the addition of 0.2, 0.4, 0.6, 0.8, and 1.0 mM DA. An oxidation peak, which corresponds to the oxidation of DA to dopaminoquinone is clearly detected at 0.45 V, indicating plasma-treated PLA is able to electrocatalyze this process. This peak, which is not observed in the absence of DA, validates the performance of plasma-treated DA as electrochemical sensors. The calibration plot obtained using the current density at the oxidation peak current, which is displayed in Figure 5b, reflects a linear behavior for the whole DA concentration range. The sensitivity, which corresponds to the slope of the calibration curve, is 0.077 mA/(cm^2^ mM) and the detection limit (DL), which refers to the lowest concentrations that can be reliably detected, is 0.27 mM.

**Figure 5:**
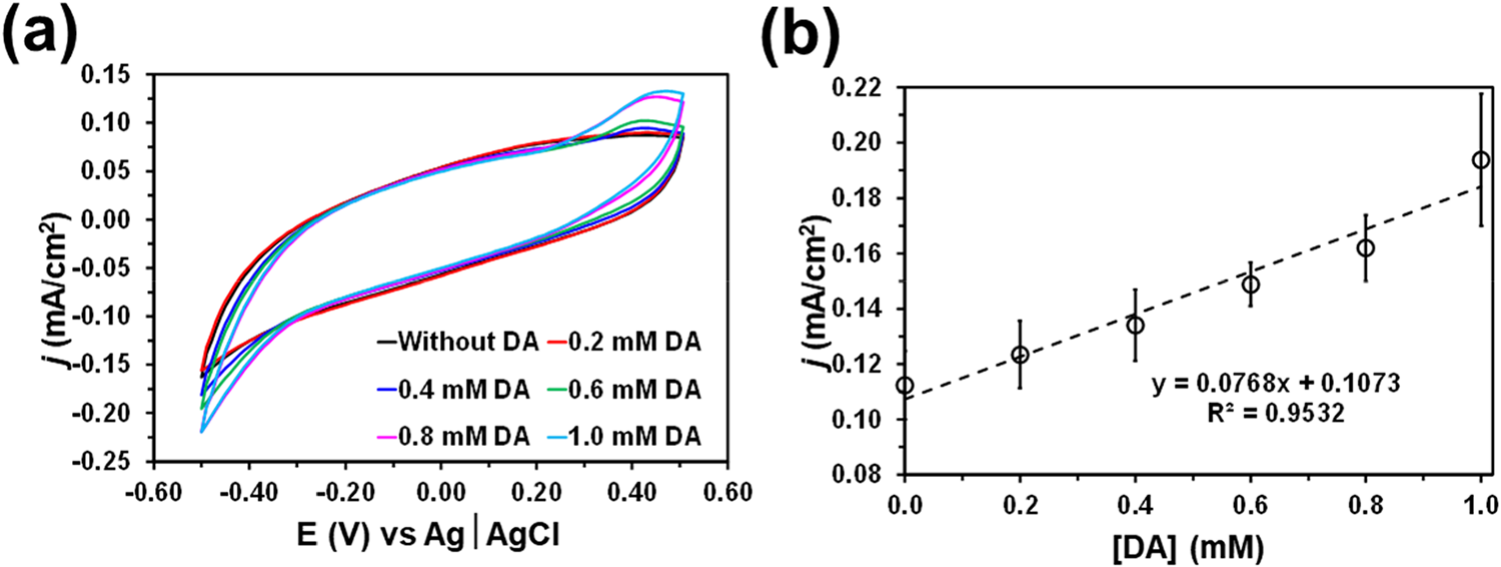
Detection of DA in PBS using PLA treated with O_2_ plasma at 0.8 mbar electrodes by CV: (a) cyclic voltammograms recorded for different DA concentrations at a scan rate of 50 mV/s and scanning a potential interval from −0.50 V (initial and final potential) to 0.50 V (reversal potential); and (b) calibration plot, which represent the peak current density of DA oxidation versus DA concentration for. Error bars show the relative standard deviation for three independent measurements.

### Computer Simulations

MD simulations on crystalline, amorphous, and plasma-treated PLA were conducted to investigate their structural differences at an atomistic level. The composition of the plasma-treated PLA chains was estimated according to the species determined by XPS for the polymer treated using an O_2_ pressure of 0.8 mbar. For this purpose, some methyl chains were replaced by hydroxymethyl and carboxylate groups, as described in the Methods Section.

Figure 6a shows the temporal evolution of the root-mean-square displacement (RMSD) considering all heavy atoms in the three studied systems. The RMSD serves as a statistical metric to quantify the molecular motion over time. As expected, the RMSD of crystalline PLA remained stable with very small fluctuations (i.e. RMSD = 0.81 ± 0.02 Å averaged over the whole trajectory), reflecting the strength of the interactions within the crystal packing. In contrast, the RMSD of amorphous PLA continuously increased over time and failed to reach a steady state even after 100 ns. This result is fully consistent with the molecular displacements expected for systems made by randomly distributed polymer chains. Furthermore, it is worth noting that the polymer chains in our model were too short to form physical entanglements. Consequently, molecular mobility predominates even below the glass transition temperature (*T*_g_ = 68 °C for PLA). Thus, in the amorphous state, PLA chains have free volume (i.e. spaces between them or at the chain ends), which helps their mobility in the absence of entanglements. Consequently, the RMSD increases from 0.81 to 1.93 Å over the 100 ns run.

**Figure 6:**
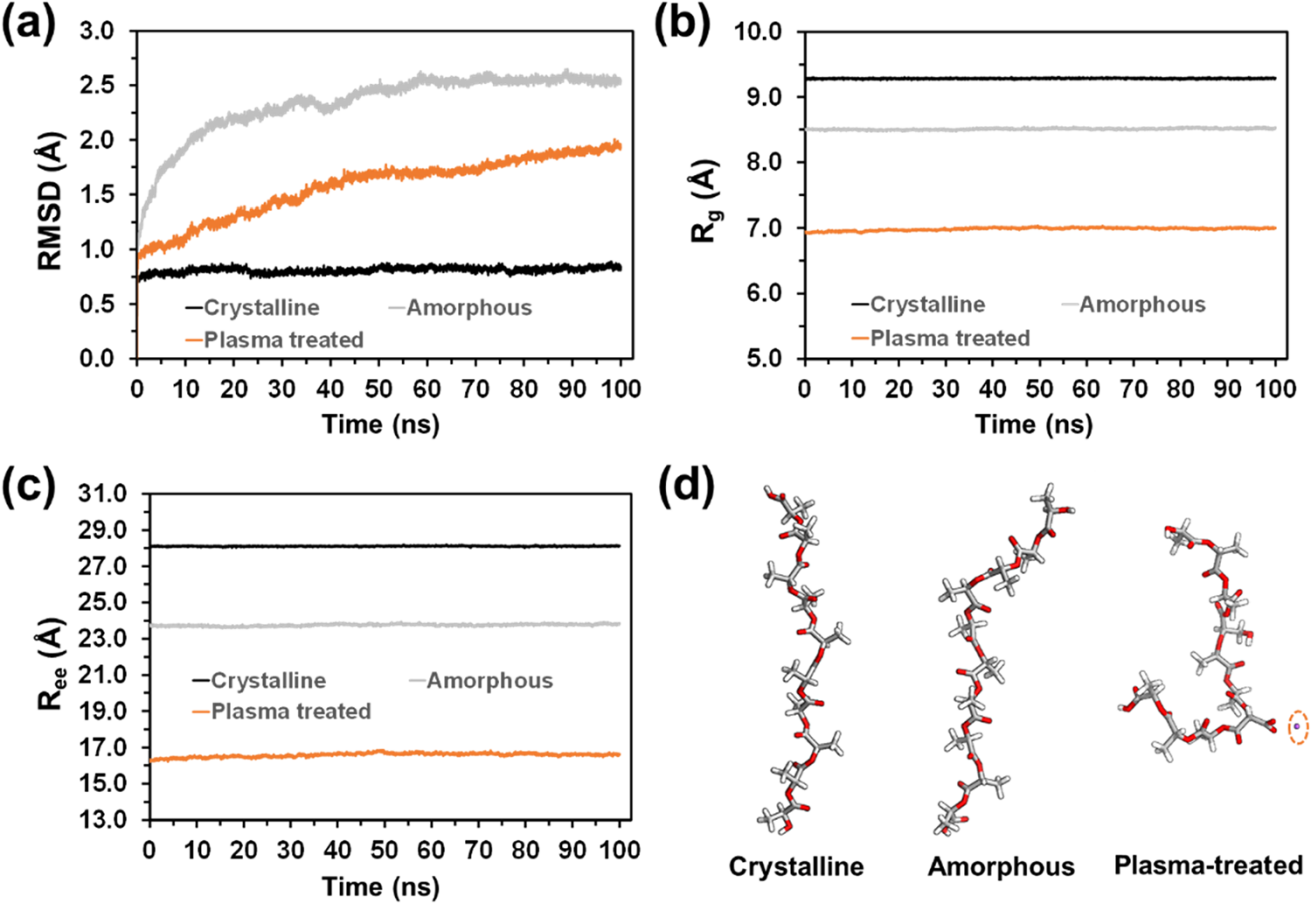
From MD simulations on crystalline, amorphous, and plasma-treated PLA: variation of the (a) RMSD, (b) *R*_g_, and (c) *R*_ee_ over time. (d) Representative polymer chains extracted from the last recorded snapshot. The position of a representative Na^+^ counterion facing the charged oxygen atoms is shown with an orange circle.

Plasma-treated PLA shows an intermediate behavior between the amorphous and crystalline PLA. Thus, the RMSD increases very rapidly during the first 15 ns (from 0.81 to 2.14 Å), and then grows slowly over the next ∼35 ns reaching a value of 2.46 Å, and finally stabilizes. Thus, the RMSD averaged over the last 50 ns is 2.54 ± 0.03 Å. This RMSD progression in plasma-treated PLA demonstrates that the polymer chains utilize free volume for movement, similar to amorphous PLA, enabling them to form sufficiently strong interactions to reach a steady state, as observed in crystalline PLA. Obviously, the formed interactions are stronger in plasma-treated PLA than in amorphous PLA because the former presents more oxygen-containing functional groups.

The average density values (ρ_cal_; in g/cm^3^) from the last 10 ns of the production MD runs are compared with experimental values (ρ_exp_; in g/cm^3^) in Table 4, demonstrating the reliability of the MD results. The error, which accounted for only 7.0%, was consistent for both the crystalline and the amorphous structures. Interestingly, both experimental measurements and computer simulations suggest a slight reduction in density after plasma treatment. This has been attributed to the incorporation of charged groups, which can form stronger attractive interactions with other groups but may also induce repulsive interactions that need to be minimized, as suggested by the time evolution of the RMSD. On the other hand, it is worth mentioning that the density predicted in this work for crystalline PLA is in good agreement with that derived using other atomistic models with larger chains (1.22 g/cm^3^).^58^ Indeed, the difference between the latter value and that displayed in Table 4 has been attributed to the end effects.

**Table 4:** Experimental and Simulated Densities for Crystalline, Amorphous, and Plasma-Treated PLA

	ρ_cal_	(ρ_exp_)	difference (%)
crystalline	1.19^a^	1.28^b^	–7.0
amorphous	1.17^a^	1.25^c^	–5.6
pristine disc	-	1.22 ± 0.03^d^	-
treated (0.4 mbar)	-	1.19 ± 0.01^d^	-
treated (0.6 mbar)	-	1.20 ± 0.001^d^	-
treated (0.8 mbar)	1.15^a^	1.19 ± 0.01^d^	–3.3

a Calculated in this work.

b From ref 47.

c From ref 59.

d Measured in this work.

Figure 6b,c shows the variation of the radius of gyration (*R*_g_) and the end-to-end distance (*R*_ee_) over time. As these are molecular properties, the represented values correspond to the averages derived from the 180 polymer chains contained in the model, which remained stable during the whole trajectories. *R*_g_ is defined as the distribution of atoms of a polymer chain around its center of mass, providing a direct measure of the compactness and size of the polymer coil. A low *R*_g_ indicates atoms are closely packed around the center, suggesting that chains tend to organize, forming disordered random structures. Conversely, a high *R*_g_ value indicates elongated chains, potentially leading to ordered aggregation (Figure 6b). Consistently, *R*_g_ is 9% higher for crystalline PLA (9.28 ± 0.01 Å) than for amorphous PLA (8.51 ± 0.01 Å). The lowest *R*_g_ corresponds to the plasma-treated PLA (6.99 ± 0.02 Å), indicating a molecular structure that is 18% more compact than that of the random coil of the amorphous state.

Similar conclusions are drawn from analyzing the temporal evolution of *R*_ee_ (Figure 6c), which represents the distance between chain ends. This parameter can vary from a maximum value, where the polymer chain is fully extended, to a minimum value corresponding to the sum of the van der Waals radii of the terminal groups. The most elongated conformation is observed in crystalline PLA (28.10 ± 0.02 Å), followed by amorphous PLA (23.76 ± 0.06 Å), whose chains are approximately 15% shorter than those of the crystalline form. Finally, the plasma-treated system exhibits the shortest *R*_ee_ (16.58 ± 0.11 Å), representing a 41% reduction in chain length compared to that of the crystalline state. Figure 6d displays a representative PLA chain taken from the final snapshot of the crystalline, amorphous, and plasma-treated simulations.

It should be noted that the stability of the polymer chain conformations, which is reflected by the practically constant compactness (*R*_g_) and molecular length (*R*_ee_) values in Figure 6b,c, supports the interpretation of the RMSD in Figure 6a. Consequently, the observed increase in RMSD in plasma-treated and, especially, amorphous systems likely results from polymer chain diffusion via translational and rotational mobility, rather than from conformational rearrangements. The very low standard deviations of the average *R*_g_ and *R*_ee_ values indicate that the conformations remained structurally stable throughout the production trajectories. This was also corroborated by the averaged dihedral angles and their standard deviations, which are shown in Table 5.

**Table 5:** Average Dihedral Angles and their Corresponding Standard Deviations for Crystalline, Amorphous, and Plasma-Treated PLA Chains

dihedral	crystalline	amorphous	plasma treated
C(H_2_)–C(HCH_3_)–C(=O)–O	78.2 ± 1.5	102.4 ± 5.2	60.3 ± 16.1
C(HCH_3_)–C(=O)–O–C(H_2_)	152.5 ± 0.3	161.2 ± 1.4	173.8 ± 3.5
C(=O)–O–C(H_2_)–C(HCH_3_)	122.3 ± 0.7	125.5 ± 1.4	131.5 ± 2.4

Radial distribution functions (RDFs) were also calculated for the three simulated systems to provide further insights into their structural properties. The RDF is an unnormalized probability distribution describing the separation (*r*) of two atoms. Figure 7a shows the RDF for the hydrogen from the OH headgroup and the oxygen from the OH tail group, denoted as g_OH‑O_(*r*). Crystalline PLA shows a sharp peak at 2.35 Å, indicative of well-ordered closest neighboring polymer molecules, and smaller, well-defined peaks at 6.65, 7.55, and 9.35 Å. The presence of these latter peaks is consistent with the lattice regularity of PLA crystalline form (Figure 8a). As expected for solid-state systems composed of short polymer chains, both the amorphous and plasma-treated systems also present a peak at a short distance (2.75 Å), which is attributed to neighboring PLA chains. Conversely, the peaks at higher distances, prominent in crystalline PLA, practically disappear, indicating a complete loss of the order and periodicity found in the crystalline state.

**Figure 7:**
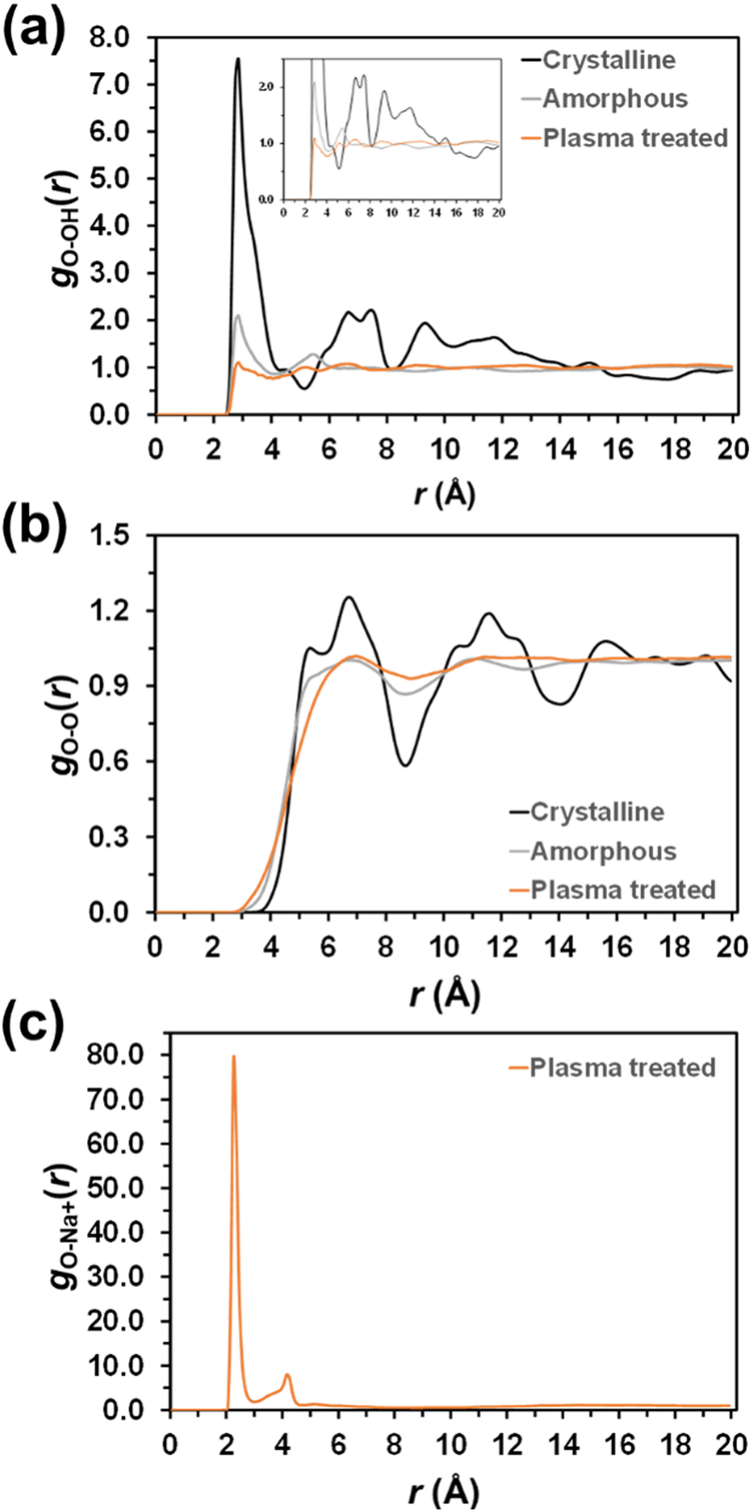
Radial distribution functions calculated for crystalline, amorphous, and plasma-treated PLA: (a) hydrogen from the OH group at the head and oxygen from the OH group at the tail, g_OH‑O_(*r*); (b) oxygen atoms belonging to different residues, g_O–O_(*r*); and (c) charged oxygen atoms and the sodium counterions in the plasma-treated structure, g_O–Na+_(*r*).

**Figure 8:**
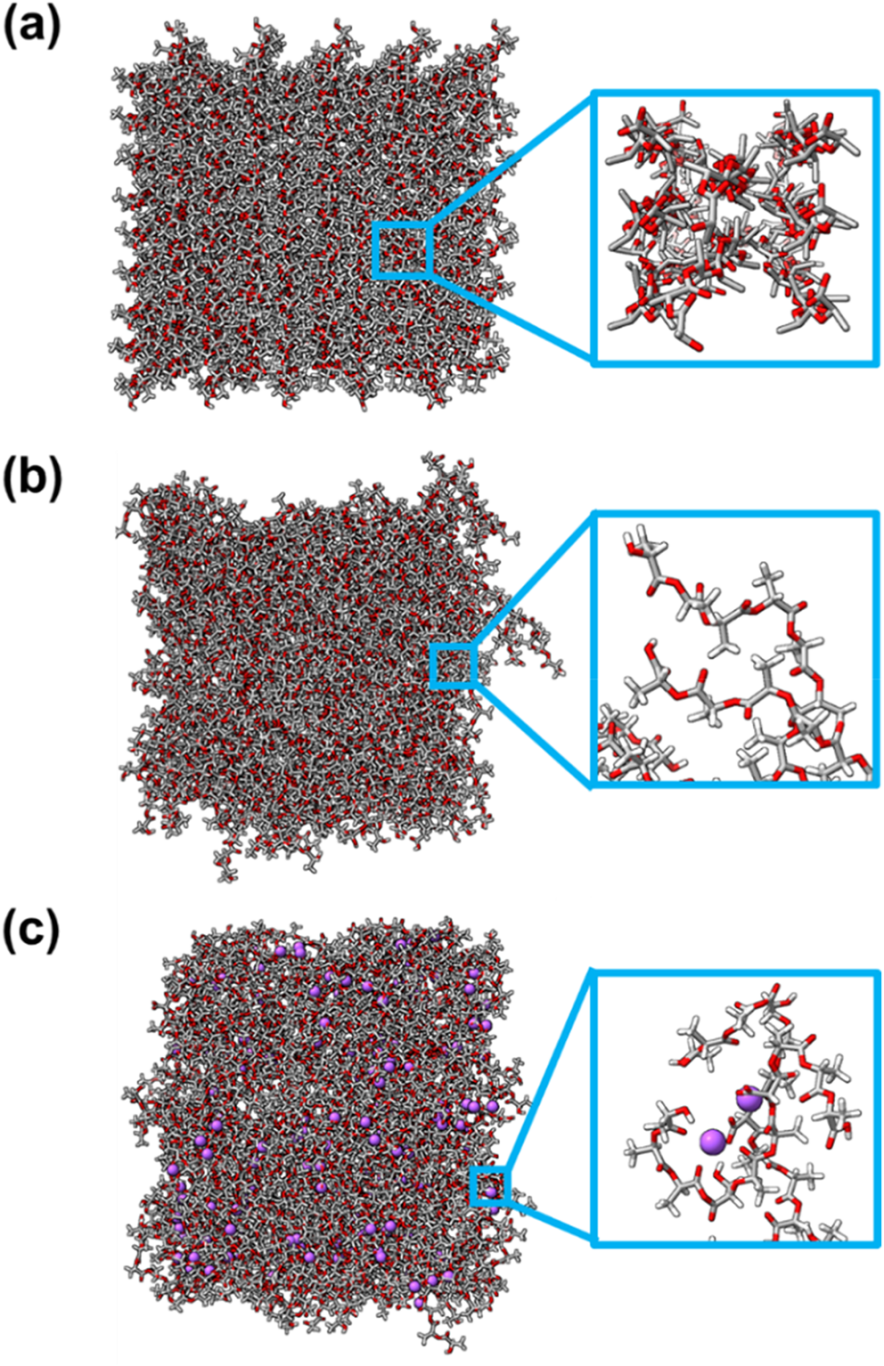
Last snapshot collected from (a) crystalline, (b) amorphous, and (c) plasma-treated PLA MD simulations. Zoom images at the right show (a) the ordered arrangement of the chain in crystalline PLA, (b) the disordered arrangement of the chain in amorphous PLA, and (c) the position of the counterions (purple color) in plasma-treated PLA.

Inspection of the RDF for oxygen atoms belonging to different residues (g_O–O_(*r*), Figure 7b) supports previous observations. While the crystalline model shows well-defined peaks, consistent with its well-defined chain conformation and 3D lattice structure, only two poorly defined peaks (6.75 and 11.05 Å) are observed for the amorphous model and just one (6.95 Å) for the plasma-treated model. This feature confirms the disordered conformation and organization of such structures, especially the plasma-treated one (Figure 8b,c). It should be noted that the temperature used for simulations was lower than the PLA glass transition temperature (*T*_g_ = 60–65 °C), which obviously also restricts the mobility of PLA chains in the amorphous phase, precluding the formation of stabilizing interactions and, therefore, the reorganization of the polymer chains.

The RDF for charged oxygen atoms and sodium counterions in the plasma-treated structure (g_O–Na+_(*r*), Figure 7c) shows a sharp, very intense peak at 2.35 Å and a smaller, well-defined peak at 4.15 Å. This indicates that the counterions directly face the charged oxygen atoms, as illustrated in Figure 8c. Despite the preferential location of Na^+^ counterions, plasma-treated PLA maintains a conformational disorder comparable to that of amorphous PLA. This is further reflected in Figure S5, which compares the RDF (g_C–C_(*r*)) for backbone carbon atoms on different chains. It is worth noting that, while crystalline PLA which displays peaks at 6.55, ∼12 and ∼17 Å, the amorphous and plasma-treated systems show no discernible peaks in their profiles.

## Conclusions

This work investigated the structural properties of 3D-printed PLA samples treated with a low-pressure O_2_ plasma. Plasma-treated PLA, widely used in food and biomedical fields, was processed using O_2_ pressures of 0.4, 0.6, and 0.8 mbar. The study yielded the following findings:

- XPS and in-depth Raman spectroscopy studies revealed the formation of oxygen-containing species within the PLA samples. Evidence included a decrease in the intensity of C–C and C–H bond fingerprints, coupled with a considerable increase in the intensity of O–C–O and O–C=O bonds. Notably, these changes were not confined to the surface but were also detected at depths up to 10 μm. Furthermore, the formation of negatively charged groups was reflected by variations in the ZP and the *R*_s_, which increased (in absolute value) and decreased, respectively. All these trends became more pronounced with increasing O_2_ pressure during plasma treatment.
- Atomistic MD simulations highlighted both structural similarities and differences between plasma-treated PLA and its crystalline and amorphous counterparts. Plasma-treated PLA chains showed a disordered conformation and organization, similar to amorphous PLA. Within this structure, neutralizing counterions were found to directly face the charged, plasma-induced oxygen-containing groups. However, plasma-treated PLA also shows a characteristic feature of crystalline PLA: a tendency to evolve toward forming strong intermolecular interactions involving the plasma-induced oxygen-containing groups. This behavior allowed the system to reach a stable steady state, similar to that observed in crystalline PLA. Overall, atomistic MD simulations have provided crucial insights, enhancing our understanding of plasma-treated PLA.
- Differences between untreated and plasma-treated PLA allow to explain the utilization of the latter as an electrochemical sensor, as reported in the literature. In order to validate such an application, the electrochemical detection of DA using plasma-treated PLA has been demonstrated, showing that such modified material is able to electrocatalyze the oxidation of DA to dopaminoquinone with a sensitivity of 0.077 mA/(cm^2^ mM) and a detection limit of 0.27 mM.

## Supplementary Material



## Notes

Atomic charges; high-resolution region of O 1s determined by XPS; ζ-potential and radial distribution functions (PDF)
