# Supplementary material for: Plasma-Treated Poly(Lactic Acid): Deciphering the Structure of a Versatile Engineering Material
Source: ACS Omega. 2025 Dec 9;10(50):61915–26. doi: 10.1021/acsomega.5c08696 (PMC12750376; doi:10.1021/acsomega.5c08696)
Supplement: Supplementary file 1 [file ao5c08696_si_001.pdf]

SUPPORTING INFORMATION

Plasma-treated poly(lactic acid): Deciphering the structure of a versatile engineering material

Adrián Fontana-Escartín,[1,2,] Nicolas Simon,[1,2] Oscar Bertran,[3] Alessandro Contini,[4] Juan Torras,[1,2,*] and Carlos Alemán[1,2,5*]*

[1] IMEM-BRT Group, Departament d'Enginyeria Química, EEBE, Universitat Politècnica de Catalunya, C/ Eduard Maristany, 10-14, 08019, Barcelona, Spain.

[2] Barcelona Research Center in Multiscale Science and Engineering, EEBE, Universitat Politècnica de Catalunya, C/ Eduard Maristany, 10-14, 08019, Barcelona, Spain

[3] Departament de Física EETAC, Universitat Politècnica de Catalunya, c/ Esteve Terrades, 7, 08860, Castelldefels, Spain

[4] Dipartimento di Scienze Farmaceutiche − Sezione di Chimica Generale e Organica "Alessandro Marchesini", Università degli Studi di Milano, Via Venezian, 21, 20133 Milano, Italy

[5] Institute for Bioengineering of Catalonia (IBEC), The Barcelona Institute of Science and Technology, Baldiri Reixac 10-12, 08028 Barcelona Spain

Page S2	**Figure S1.** Atomic charges.<br>**Figure S2.** High resolution region of O 1s and decomposed peaks as determined by XPS.
Page S3	**Figure S3.** Raman spectra at depths of 1, 5 and 10 μm.
Page S4.	**Figure S4.** Variation of the Zeta-potential with the O_2 pressure.<br>**Figure S5.** Radial distribution functions for carbon atoms belonging to different chains.

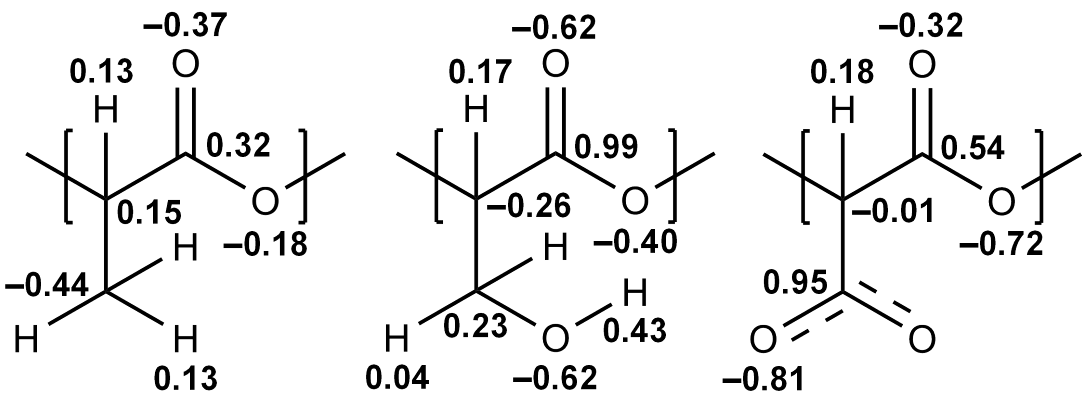

Figure S1. Atomic charges used for MD simulation of amorphous and crystalline PLA, and plasma-treated PLA.

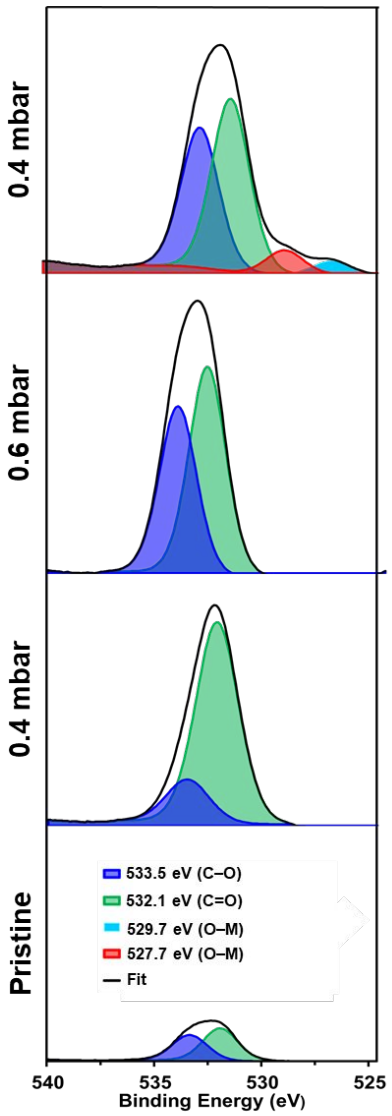

Figure S2. High resolution region of O 1s and decomposed peaks for pristine and plasma-treated PLA samples as determined by XPS.

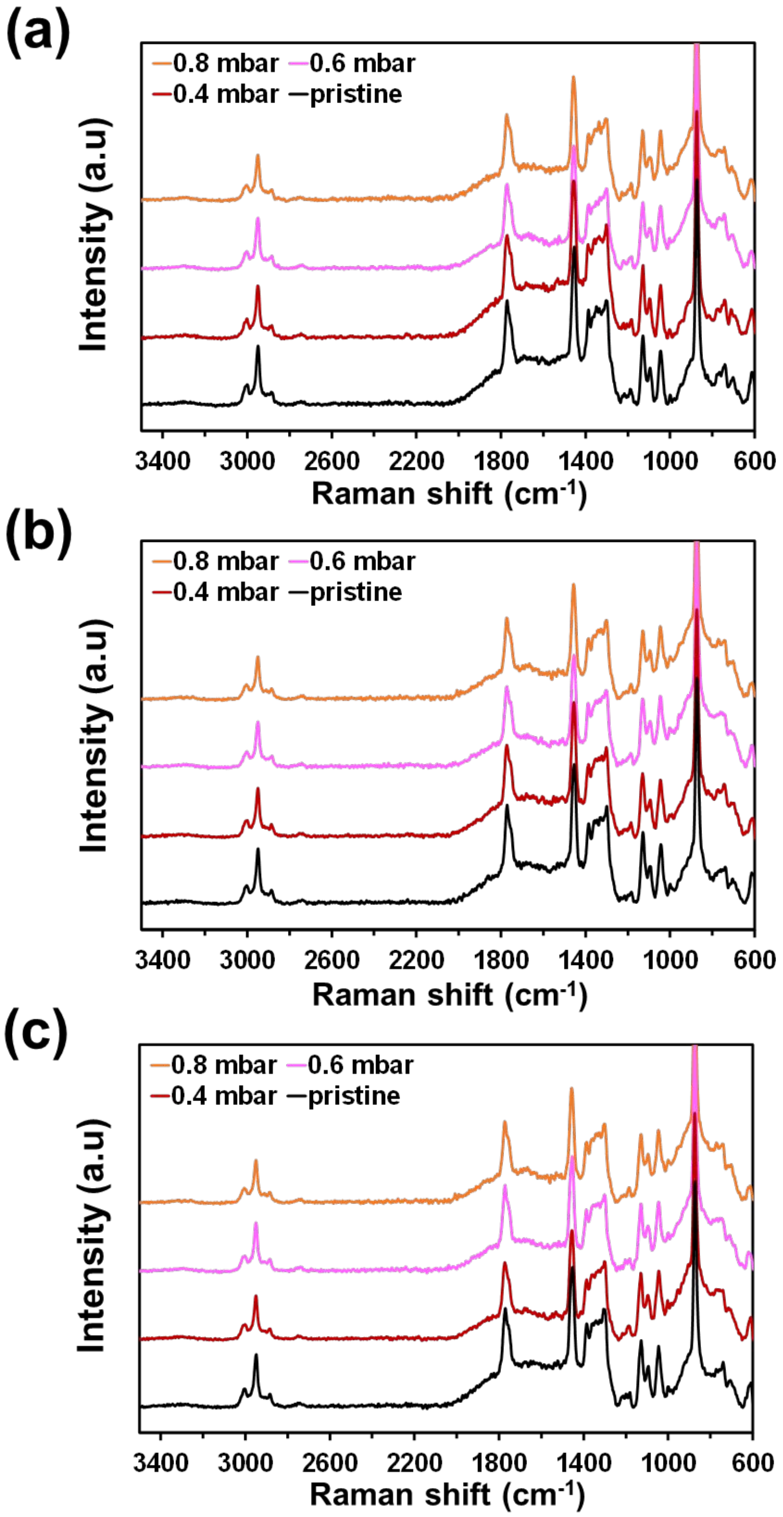

Figure S3. Raman spectra of pristine and plasma-treated PLA at depths of (a) 1, (b) 5 and (c) 10 μm.

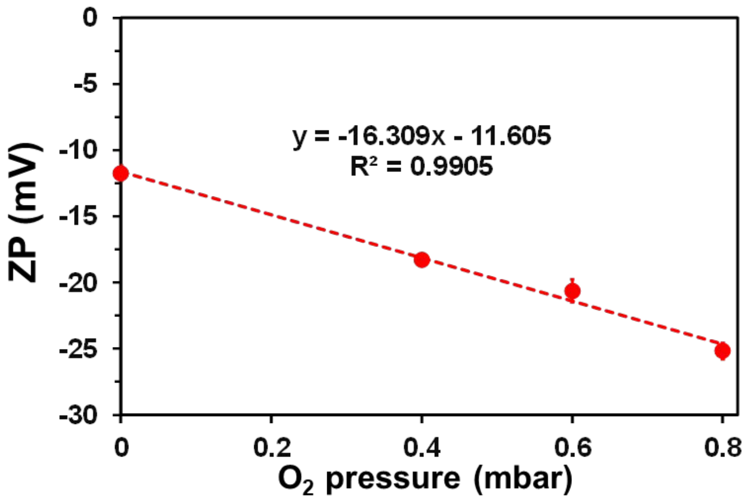

Figure S4. Variation of the Zeta-potential (ZP) with the O_2 pressure used for the PLA plasma treatment. Pressure of 0 mbar corresponds to pristine PLA (*i.e.* non-treated PLA).

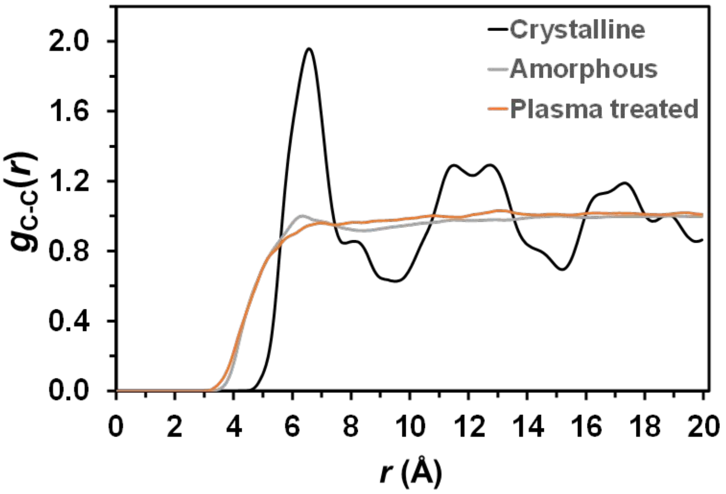

Figure S5. Radial distribution functions calculated for crystalline, amorphous and plasma-treated PLA: carbon atoms belonging to different chains, $g_{C\text{-}C}(r)$.
